# Humoral immune responses in different stages of wound healing in Black Bengal goats

**DOI:** 10.5455/javar.2023.j688

**Published:** 2023-09-24

**Authors:** Kanika Samaddar, Mohammad Habibur Rahman, Md. Leonur Islam Leon, Md. Sohel Rana, Md. Mehedi Hasan, Ziaul Haque, Md. Mizanur Rahman

**Affiliations:** 1 Department of Surgery and Obstetrics, Bangladesh Agricultural University, Mymensingh, Bangladesh; 2 Immunology and Vaccinology Laboratory, Department of Microbiology and Hygiene, Bangladesh Agricultural University, Mymensingh, Bangladesh; 3 Department of Anatomy and Histology, Bangladesh Agricultural University, Mymensingh, Bangladesh

**Keywords:** Humoral immune responses, wound healing, ELISA, Igs, IL-6, black Bengal goat

## Abstract

**Objective:**

The current study was carried out to assess the humoral immune responses according to age at different stages of wound healing in Black Bengal goats (BBG).

**Materials and Methods:**

Apparently, healthy BBGs (*n* = 20) were collected and divided into five groups based on their age: Group A (control, 3 years), Group B (3 to 5 years), Group C (2 to <3 years), Group D (1 to <2 years), and Group E (<1 year). Except for control, all BBGs were allowed to have artificial surgical wounds, and follow-up data were collected from day 0 to 21. The humoral immune responses [immunoglobulins (Igs) and interleukin-6 (IL-6)] were determined by ELISA using commercial goat ELISA kits. Statistical Product and Service Solutions (Version 20) was used to analyze the data.

**Results:**

The normal range of immune cells in control BBGs was immunoglobulin G (IgG) (20.21 ± 0.13 mg/ml), immunoglobulin M (IgM) (2.87 ± 0.0.05 mg/ml), immunoglobulin A (IgA) (0.33 ± 0.01 mg/ml), and IL-6 (1.6 ± 0.05 pg/ml). In this experiment, higher concentrations of IgG (21.11 ± 0.20 mg/ml), IgM (2.92 ± 0.04 mg/ml), IgA (0.35 ± 0.02 mg/ml), and IL-6 (1.62 ± 0.05 pg/ml) were found in Group B BBGs, whereas the lower levels of IgG, IgM, IgA, and IL-6 were found at 17.16 ± 0.18 mg/ml, 2.12 ± 0.01 mg/ml, 0.29 ± 0.03 mg/ml, and 1.55 ± 0.05 pg/ml, respectively, in the Group E BBGs. Rapid wound healing was observed in the older groups compared to the younger groups of BBGs. The concentrations of Igs (IgG, IgM, and IgA) and IL-6 were gradually increased in all groups from day 3 (early inflammatory stage) and day 7 (late inflammatory stage), and then they decreased gradually from day 14 (proliferative stage) to reach the final stage of day 21 (remodeling stage), where the concentrations were found to be at a level comparable to their per-incisional period. No gender-related differences were detected.

**Conclusion:**

Adult BBGs (3 to 5 years old) showed faster wound repair and stronger immune responses. This finding may assist veterinarians and researchers in considering age-related immune responses for the recovery and rapid cure of surgical wounds.

## Introduction

Goats have always been an important part of mixed farming practices in Bangladesh and supply 38.0%, 23.0%, and 28.0% of meat, milk, and skin, respectively, in the livestock sector [1]. Most rural communities in Bangladesh earn a significant part of their income from goat rearing. Goats are thought to be the best type of livestock because they can thrive in extreme conditions and produce optimum-quality milk and meat [2]. Black Bengal goats (BBGs) are well-known for their adaptability, fertility, meat test, skin, adaptability in harsh conditions, and disease resistance [3].

The host immune system is the body’s protective mechanism, comprised of both adaptive and innate immunity, which aids in the fight against toxic or allergic agents, diseases, and pathogenic microbial infections in conjunction with cytokines and growth factors [4,5]. Humoral immunity is the adaptive immune system that controls macrophages, facilitates fibroblastocysts, and neutralizes harmful bacteria and viruses [6,7]. It is an antibody-mediated [immunoglobulin A (IgA), immunoglobulin G (IgG), immunoglobulin M (IgM), immunoglobulin D, and immunoglobulin E) response against pathogens triggered by vaccinations or diseases that is important in host immune defense [8,9].

A wound is the discontinuity of skin, muscles, and tissues caused by physical, chemical, or biological insults [10]. Wound healing is a dynamic and highly regulated cellular, humoral, and overlapping complex biological process for restoring the integrity of the skin after injury [11]. When the skin is cut, it sets off a chain of inflammatory processes that quickly bring phagocytes from the bloodstream to the wound site [12]. During different stages of wound repair, multicellular organisms display immunological homeostasis in various cells and organs with steps like coagulation, inflammation, and proliferation [13,14].

Among the pro-inflammatory cytokines, interleukin-6 (IL-6) is crucial for adaptive and innate immune function [15,16]. IL-6 also plays a significant role in stimulating the proliferation of antibody-producing B cells, resulting in an increased immunological response and cutaneous wound healing [15,17]. During inflammatory mechanisms, the concentration of IL-6 multiplies, emphasizing its clinical significance as a primary alarm system in inflammation and autoimmune disease in goats [16,18]. A healthy immune system can speed up wound healing by lowering the infection risk. Wound healing remains challenging when humoral immune responses are reduced [19]. Therefore, considering the importance of age-related immune responses in goats, the current study investigated age-related humoral immune responses at different phases of wound repair in BBGs in Bangladesh.

## Materials and Methods

### Ethical approval

The experimental protocols were evaluated and approved by the Animal Welfare and Experimentation Ethics Committee, Bangladesh Agricultural University, Mymensingh [AWEEC/BAU/2019(54)]. Principles were tracked to afford slight distress to the experimental BBGs to reduce the perception of pain; local analgesia was administered before wound initiation.

The current study was conducted from June 2020 to 2022 in different laboratories of the Department of Surgery and Obstetrics, the Department of Microbiology and Hygiene, and the Department of Anatomy and Histology, Bangladesh Agricultural University.

### Selection and management of experimental goats

Twenty healthy BBGs of different ages were randomly selected and purchased from the local markets of Mymensingh, Bangladesh. The experimental goats were reared at the Department of Surgery and Obstetrics goat shed under well-supervised conditions and fed the same diet. All the goats were dewormed against internal parasites with a broad-spectrum anthelmintic (Endex^®^) and external parasites with A-Mectin Plus Vet and vaccinated against tetanus (Tetanus Vaccine^®^) and Peste des Petits Ruminants (PPR-Vac^®^). Following adaptation for 3 weeks, the BBGs were allowed to graze on pasture land for approximately 8 h daily, along with 300 gm of formulated concentrates per goat per day and free access to drinking water. Regular deworming at 4-month intervals and vaccination against PPR were performed.

### Experimental design

The experimental BBGs were equally distributed into five groups based on age: Group A (3 to 3.5 years of age) as a control for analyzing the normal values of immunoglobulins (Igs) and IL-6 in BBGs; Group B (3 to 5 years); Group C (2 to >3 years); Group D (1 to >2 years); and Group E (<1 year).

### Artificial surgical wound introduction in BBGs

A single dose of intramuscular injections of 6 mg/kg xylazine hydrochloride (Xylaxin^®^, 23.22 mg/ml) and 50 mg/kg ketamine hydrochloride (G-Ketamine^®^, 50 mg/ml ketamine hydrochloride USP) was used to sedate the goats. Following anesthesia, except for control BBGs, the back region was shaved and cleaned with a 10% solution of povidone-iodine (Povisep^®^), two surgical lesions of 3 cm in length and 0.5 cm in depth were created by a vertical incision, the skin was detached from the underlying tissues, and the lesions were sealed with a simple discontinuous silk stitch. All sewing was separated by 8 mm; there was a 5 mm gap between the needle location and the cutting edge. All lesions were sealed using nylon and three throws of cross-mattress suture. All goats were kept under regular observation, and good feed was provided two times per day after surgery was performed.

### Blood collection, serum preparation, and analysis

To determine the Igs and IL-6, about 5 ml of blood were collected from each goat of the five groups on days 0 (just before lesions were created), 3 (D3), 7 (D7), 14 (D14), and 21 (D21) by sterile vacutainers, and sera were separated by centrifugation at 3,500 rpm for 10 min and stored at −20°C. The collected sera were analyzed by ELISA using commercial goat Igs and IL-6 kits to analyze the concentration of Igs and goat IL-6 to determine the humoral immune responses in different stages of wound healing in BBGs.

### Statistical analysis

An independent sample *t*-test was conducted using Statistical Product and Service Solutions version 20.0 to compare group data. The effects of age on the immunological profiles of BBGs in different stages of wound healing were analyzed by one-way analysis of variance, and the Duncan multiple range test was applied for post hoc comparison using the general linear model.

## Results

In this study, humoral immune responses at different stages of wound healing in experimental BBGs were determined by evaluating Igs (IgG, IgM, and IgA) and IL-6 levels in collected sera.

### Ig levels in control (Group A: 3 years) BBGs

**Figure 1.**
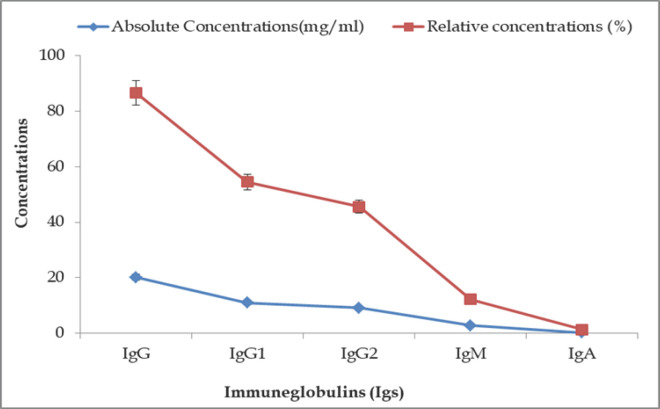
Graphical representation of different Igs concentrations in control BBGs.

This study revealed the normal levels of IgG, IgM, and IgA in BBG to be 0.33 ± 0.01, 2.87 ± 0.05, and 20.21 ± 0.13 mg/ml, respectively (Figure 1). The relative levels of IgA, IgM, and IgG concentrations were 1.41%, 12.26%, and 86.57%, respectively, in adult healthy control BBGs (Group-A). The absolute (20.21 ± 0.13 mg/ml) and relative concentrations (86.57%) of IgG were comparatively higher than other Igs. The absolute and relative concentrations of IgA (0.33 ± 0.01 mg/ml and 1.41%) were found in the control BBG serum. In BBGs, two subtypes of IgG were found: IgG1 (54.43%) and IgG2 (45.57%). During the immune response, IgG1 (11.01 ± 0.07) increased sharply, whereas variations in IgG2 concentrations (9.20 ± 0.06) were less evident.

### Age-related Igs (IgA, IgG, and IgM) as humoral immune response in BBGs

**Figure 2.**
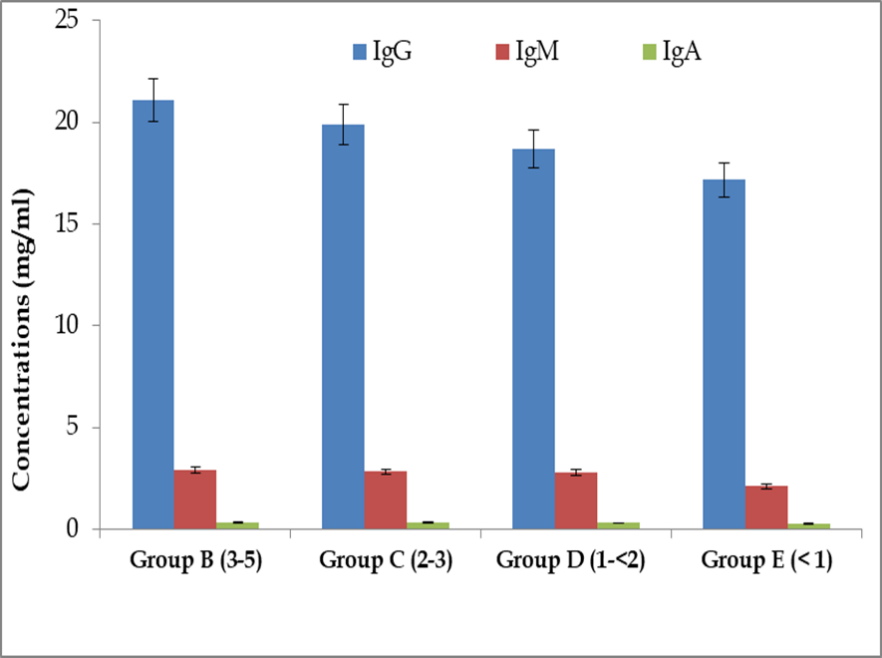
Age-related IgG (mg/ml) response in different groups of BBGs.

Several age-related alterations in the examined immunological parameters were detected in the current investigation. The greater levels of IgM, IgG, and IgA were found at 2.92 ± 0.04, 21.11 ± 0.20, and 0.35 ± 0.02 mg/ml, respectively, in the goats of Group B. On the other hand, the lower values of IgA, IgG, and IgM were 0.29 ± 0.03, 17.16 ± 0.18, and 2.12 ± 0.01 mg/ml, respectively, in Group E BBGs. The IgG concentrations of Group B (21.11 ± 0.20 mg/ml), Group C (19.91 ± 0.19 mg/ml), and Group D (18.69 ± 0.19 mg/ml) were significantly (*p *< 0.01) higher than Group E (17.16 ± 0.18 mg/ml). Still, no significant difference was found between Group B and Group C or between Group C and Group D. The IgM concentration of Groups B, C, and D was significantly (*p *< 0.01) higher than that of Group E. Still, no significant difference was found between Groups B, C, and D. The IgA concentration of Groups B and C was significantly (*p *< 0.05) higher than that of Group E, but no significant difference was found between Groups B and C or between Groups D and E (Figure 2).

### Age-related alteration of IgG response in different stages of wound healing in BBGs

**Figure 3.**
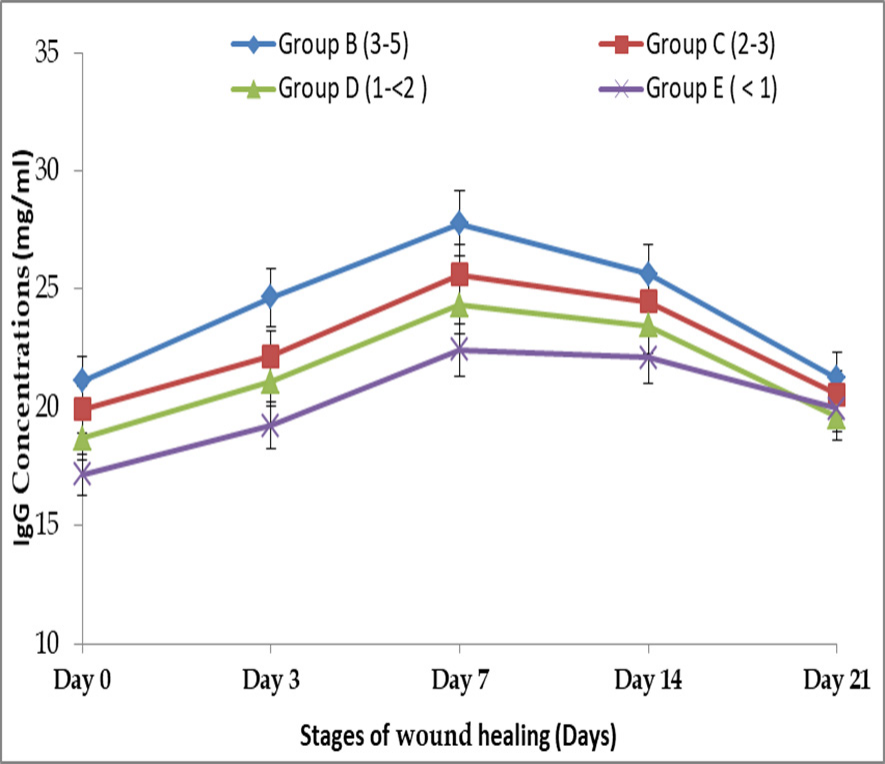
Change in IgG (mg/ml) response in different stages of wound healing in BBGs.

Age-related variations of IgG were observed in different stages of wound healing in Group B on days 0, 3, 7, 14, and 21, and the concentrations were 21.11 ± 0.20, 24.65 ± 0.16, 27.78 ± 0.14, 5.63 ± 0.10, and 21.24 ± 0.05 mg/ml, respectively. IgG concentrations in Group C BBGs were found to be 19.91 ± 0.19, 22.15 ± 0.16, 25.61 ± 0.14, 24.46 ± 0.11, and 20.55 ± 0.11 mg/ml, respectively, on the same days. IgG concentrations in Group D were found to be 18.69 ± 0.19, 21.09 ± 0.22, 24.32 ± 0.15, 23.44 ± 0.10, and 19.58 ± 0.09 mg/ml, respectively. In Group E, 17.16 ± 0.18, 19.24 ± 0.15, 22.43 ± 0.15, 22.11 ± 0.07, and 19.95 ± 0.14 mg/ml were correspondingly found on the same days of wound healing (Figure 3). Here, the IgG concentrations at day 0 were significantly (*p *< 0.01) higher in Groups B, C, and D than in Group E, but no significant difference was found between Groups B and C or between Groups C and D. The IgG concentrations on days 3, 7, and 14 were significantly (*p *< 0.01) higher in Group B than in the rest of the groups. The IgG concentrations on day 21 were significantly (*p *< 0.01) higher in Group B than in Groups D and E. Still, no significant differences were found between Groups B and C, between Groups C and E, or between Groups D and E.

### Age-related alteration of IgM response in different stages of wound healing in BBGs

**Figure 4.**
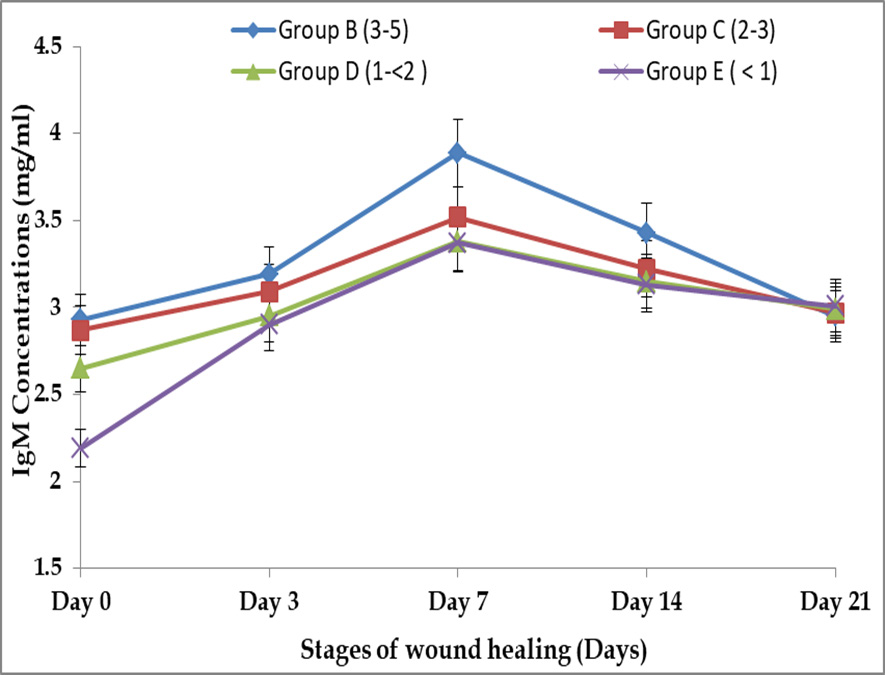
Age-related IgM response in different stages of wound healing in BBGs.

The IgM (mg/ml) response in different phases of wound repair in BBGs was found to be 2.93 ± 0.05, 3.19 ± 0.07, 3.89 ± 0.08, 3.43 ± 0.10, and 2.95 ± 0.12 mg/ml, respectively, in Group B on days 0, 3, 7, 14, and 21. Whereas, in Group C, the concentrations were found to be 2.87 ± 0.03, 3.09 ± 0.10, 3.52 ± 0.09, 3.22 ± 0.07, and 2.97 ± 0.05 mg/ml, respectively. In Group-D, IgM was found to be 2.65 ± 0.03, 2.95 ± 0.07, 3.38 ± 0.09, 3.15 ± 0.04, and 2.99 ± 0.05 mg/ml, and in Group E, we observed IgM of 2.19 ± 0.07, 2.90 ± 0.07, 3.37 ± 0.09, 3.13 ± 0.05, and 3.01 ± 0.02 mg/ml, respectively, at the same stages of wound healing. IgM concentrations at different days were not significantly (*p *> 0.05) different between groups. In this experiment, it was found that the IgM concentration increased at different stages of postoperative wound healing. Goats aged 3 to 5 years (Group B) showed the most rapid changes in IgM concentration in different stages. On the other hand, the goats in Group D exhibit slower changes in IgM concentration and delayed wound healing. The serum IgM concentrations were increased to their highest on the 7th day and then decreased from the 14th day to the 21st day in level toward the same pattern that was displayed in pre-incisional time (day 0), which is shown in Figure 4.

### Age-related alteration of IgA concentrations in different stages of wound repair in BBGs

**Figure 5.**
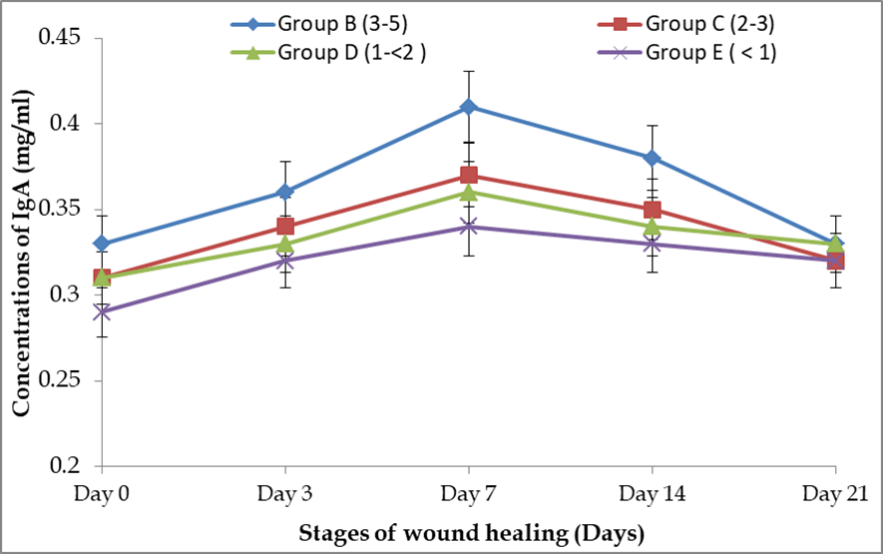
Age-related IgA response in different stages of wound healing in BBGs.

The IgA concentrations in different phases of wound repair in Group B BBGs were found to be 0.33 ± 0.005, 0.36 ± 0.005, 0.41 ± 0.005, 0.38 ± 0.005, and 0.33 ± 0.004 mg/ml, respectively, on days 0, 3, 7, 14, and 21. Whereas, Group C showed 0.31 ± 0.006, 0.34 ± 0.008, 0.37 ± 0.004, 0.35 ± 0.003, and 0.32 ± 0.004; Group D showed 0.31 ± 0.007, 0.33 ± 0.007, 0.36 ± 0.004, 0.34 ± 0.005, and 0.33 ± 0.006 mg/ml, respectively; and Group E exhibited 0.29 ± 0.005, 0.32 ± 0.005, 0.34 ± 0.003, 0.33 ± 0.004, and 0.32 ± 0.004 mg/ml, respectively, on the same days (Figure 5). There were no significant differences in IgA concentrations at days 0, 3, and 21 in different groups. The IgA concentration was significantly (*p *< 0.01) higher in Group B than in other groups on day 7. The IgA was significantly higher in Groups B and C than in Groups D and E. Still, no significant (*p *< 0.05) difference was found between Groups B and C or among Groups C, D, and E. Like IgG and IgM, the amount of IgA is also high in older groups.

### Changes in IL-6 (pg/ml) concentration of BBGs according to age

**Figure 6.**
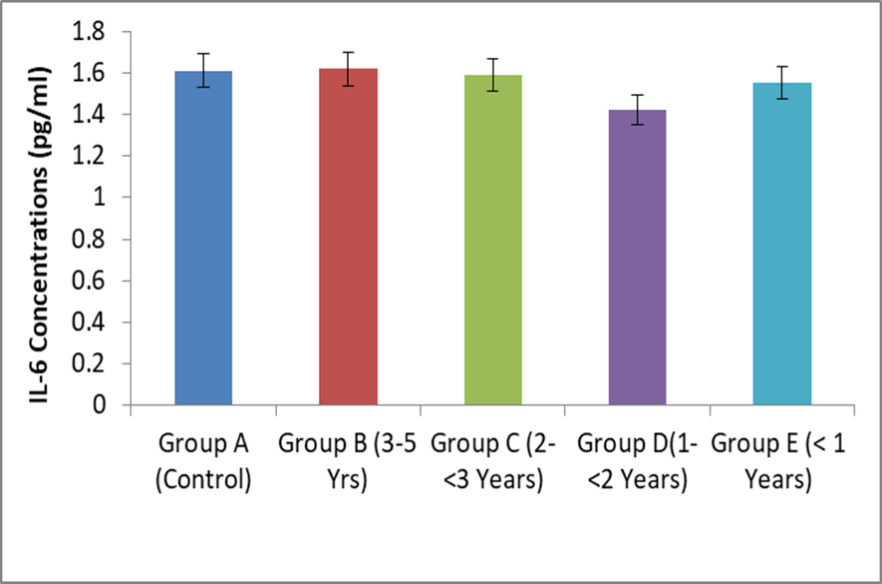
Age-related IL-6 response in BBGs.

Among the pro-inflammatory cytokines, IL-6 plays a key role in the humoral and cell-mediated immune systems of BBGs. The higher concentrations (1.62 ± 0.05 pg/ml) were found in Group B goats, and the lower concentrations (1.55 ± 0.04 pg/ml) were found in Group E goats (Figure 6).

### IL-6 response in different stages of wound healing in BBGs

**Figure 7.**
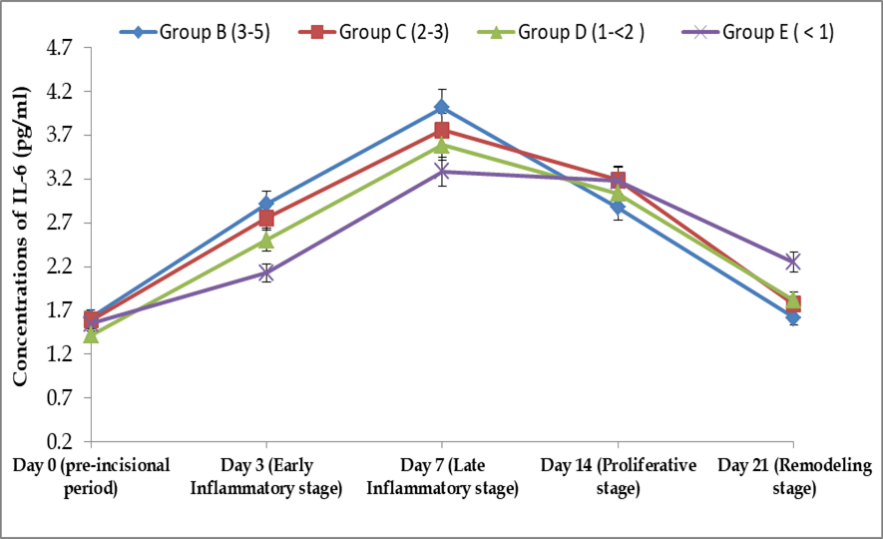
Age-related IL-6 (pg/ml) response in different stages of wound healing in BBGs.

The serum IL-6 response in Group-B BBGs was found to be 1.62 ± 0.05, 2.92 ± 0.05, 4.02 ± 0.05, 2.88 ± 0.04, and 1.62 ± 0.04 pg/ml at days 0, 3, 7, 14, and 21, respectively. In Group C, the IL-6 was found at 1.59 ± 0.04, 2.76 ± 0.06, 3.76 ± 0.04, 3.19 ± 0.07, and 1.77 ± 0.05 pg/ml, respectively; in Group D, we observed 1.42 ± 0.05, 2.51 ± 0.05, 3.59 ± 0.07, 3.04 ± 0.04, and 1.82 ± 0.06 pg/ml, respectively, on the same days. In Group E, it was found at 1.55 ± 0.04, 2.13 ± 0.06, 3.29 ± 0.03, 3.18 ± 0.05, and 2.25 ± 0.08 pg/ml, respectively, at 0, 3, 7, 14, and 21 days post-infection (Figure 7). The IL-6 concentrations were increased to their highest levels on day 7, and then gradually decreased from day 14 up to day 21.

## Discussion

In this experiment, it was found that Igs (IgG, IgM, and IgA) concentrations were increased at different stages of the postoperative days of wound healing. The BBGs of different ages showed various levels of Igs (IgG, IgM, and IgA) according to their humoral immune response to wound healing. In the present age-related humoral immune response experiments, the normal levels of Igs (IgG, IgM, and IgA) in healthy adult (Group-A) BBGs were found to be 20.21 ± 0.13, 2.87 ± 0.05, and 0.33 ± 0.01 mg/ml, respectively. These findings were within the standard limit and supported by Pomorska-Mól and Markowska-Daniel [20] and Donovan et al. [21]. However, the Igs level was much lower than the report of Bayram et al. [22], and minor discrepancies were found with the findings of Pomorska-Mól et al. [23]. This investigation demonstrated that, irrespective of the goat’s age, IgG was the main Igs in the serum. In contrast, IgA constituted the smallest class of serum Igs identified in goat serum. According to Herr et al. [24], Igs as glycoproteins are essential components of the humoral immune system. Three classes of antibodies have been found, and their prevalence in the blood serum is in the order of IgG>IgM>IgA.

In the current study, the concentrations of IgA, IgM, and IgG in sera rose with age, with the highest amounts reported in goats between 3 and 5 years old, which displayed rapid wound healing. Similar findings were also reported: older individuals have a higher IgG concentration (both absolute and relative) than younger individuals [21,25,26]. As the immune system grows, Igs production becomes more efficient, and serum IgG, IgM, and IgA concentrations increase [27]. The goats under 1 year showed lower IgG, IgM, and IgA concentrations and a slower wound healing process. This is due to the lack of maturity of the lymphoid system [27]. According to Flaminio et al. [28], the serum IgM and IgG concentrations of goats grew proportionally with their ages, and age-related variations in humoral and cell-mediated development were similarly observed in horses [29]. Therefore, age is an essential factor in evaluating serum Igs content. Here it was found that IgG, IgM, and IgA concentrations were more or less increased to their highest level on days 7 and 14, then decreased to levels nearer to their normal range on day 21 in BBGs; similar observations were agreed upon by the findings of Aschermann et al. [30].

The IL-6 concentration (1.60 pg/dl) found in this experiment was close to the finding of Effros et al*.* [31] but considerably lower than that described by Roubenoff et al. [32]. The concentration of IL-6 in serum increased with the age of BBGs, with a higher level observed in the 3–5-year-old age group, and similar results were reported in previous studies [31,32]. The concentration of IL-6 significantly increased on day 7, as shown in Figure 6. In 1996, Tsujinaka et al. [33] reported that IL-6 levels are raised in various inflammatory conditions and, in experimental animals, can generate cachexia. Adult BBGs (3–5 years old) showed higher concentrations and rapid wound healing in response to IL-6. Besides, the BBGs under 1 year showed a lower concentration of IL-6 and delayed wound healing. These findings were due to the maturation of the lymphoid system and, consistent with previous research, the elevated serum IL-6 levels in apparently healthy elderly subjects but not in younger animals [31,34,35].

In the current investigation, the concentrations of Igs (IgG, IgM, and IgA) and IL-6 differed significantly (*p *< 0.01) among BBGs of different ages; this effect was partially supported by the study of Shaikat et al*.* [36]. The increase in immune concentrations may be attributed to the acute inflammation evocated by the wound, and the Igs and IL-6 values were gradually restored to their normal pre-incisional levels at 21 days. The concentrations of Igs and IL-6 were elevated in the first week of post-wounding in all the groups of BBGs with significant differences; these findings are consistent with the study of Crovetti et al. [37]. As this is the first report in Bangladesh for age-related immune responses in BBGs, further study is needed considering species, sex, seasonality, and environmental variables for a better understanding of immune responses in goats.

## Conclusion

In a nutshell, it can be concluded that both the relative and absolute serum Igs and IL-6 concentrations are affected by age. All Igs concentrations were higher in older BBGs than younger ones. Therefore, the older group showed a faster wound-healing process. The current study depicted age-related immunological variables in terms of the physiological development system, lack of maturity of the immune function, and immunological reactions within the body throughout normal development. The current findings also suggest that valid comparisons of serum Igs levels (relative and absolute) between diseased and healthy animals are impossible without sufficient regulation of age-related biological changes. The findings will also assist veterinarians with the molecular pathways by which the immune system regulates the wound healing process at different ages, thereby facilitating the development of novel regenerative therapies.

## List of abbreviations

BBGs, Black Bengal Goats; °C, Degree Celsius; DMRT, Duncan Multiple Range Test; hrs., Hour; IgA, Immunoglobulin A; IgG, Immunoglobulin G; IgM, Immunoglobulin M; IL-6, Interleukin-6.
